# Evolutionary History and Climatic Correlates of Hypermelanism in Viperidae

**DOI:** 10.1002/ece3.71305

**Published:** 2025-04-16

**Authors:** Federico Storniolo, Marco Mangiacotti, Marco A. L. Zuffi, Stefano Scali, Roberto Sacchi

**Affiliations:** ^1^ Dipartimento di Scienze della Terra e dell'Ambiente Università degli Studi di Pavia Pavia Italy; ^2^ Museo di Storia Naturale dell'Università di Pisa Calci Italy; ^3^ Museo di Storia Naturale di Milano Milano Italy

**Keywords:** Bogert's rule, ecology, evolution, phylogeny, vipers

## Abstract

Body colorations have been investigated intensely concerning their adaptive significance from the ecological and evolutionary perspectives. Studies on melanism have caught growing interest thanks to its marked variability across space, time and taxon and, in ectotherms, it has been hypothesised to be driven by thermal advantages. Among reptiles, vipers show conspicuous inter‐ and intraspecific patterns of variation, making them excellent models to address evolutionary and adaptive patterns. We investigated the thermal melanism hypothesis across Viperidae by performing a phylogenetic comparative approach to assess whether its occurrence is phylogenetically driven or, alternatively, whether it is influenced by climate. Phylogenetic signal was detected and reconstructed the ‘non‐melanistic’ form as the ancestral state at the root of their phylogeny, whereas a climatic effect was found so that melanism is more frequent in colder environments. With this work, we provide on a large geographical scale strong support for the putative advantages provided by melanism in colder climates; moreover, melanism appears to have evolved in multiple events throughout the diversification of vipers, but it has been rarely maintained over time. We hypothesise that it is maintained only when environmental conditions, such as low thermal regimes, render it favourable; nevertheless, experimental evidence is necessary to further support this hypothesis.

## Graphical abstract

We performed a two‐step analysis on the occurrence of melanism in Viperidae: Firstly, Ancestral State Reconstruction was performed so that the non‐melanic state was estimated as the most probable at the root of the vipers' phylogenetic tree; secondly, we tested the Thermal Melanism Hypothesis on the spatial occurrence of melanism in this snake family, showing that it is negatively correlated with average diurnal temperatures. The evolutionary history of melanism in this group appears to have followed a complex pattern of acquisitions and losses, and we hypothesise that this pattern is related to climatic effects.

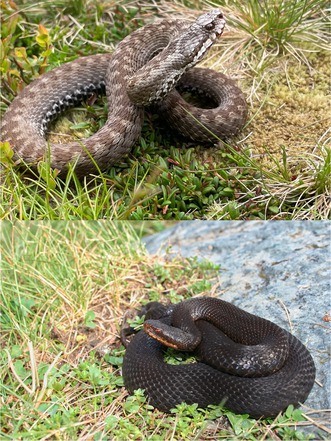

## 1 Introduction

In animals, body colorations are markedly informative of health condition (Halliday et al. 2014), reproductive status (Vercken et al. 2007) and involved in social interactions with conspecifics (Lebas and Marshall 2000; Ogilvy et al. 2012). Colorations are also frequently under selection for camouflage (Cuadrado et al. 2001; Marshall et al. 2016) and, in ectotherms such as reptiles, for thermoregulation (Smith et al. 2016); therefore, the evolution of body colourations must be investigated by accounting for several aspects of species' evolutionary history and adaptive processes. Melanism (i.e., an increased amount of dark, melanin‐based integument due to increased melanogenesis; True 2003) is one of the most investigated types of coloration and is ubiquitous across vertebrates. For instance, the occurrence of melanistic individuals among mammals has been known for long time especially in felids and rodents (Majerus and Mundy 2003; Schneider et al. 2015) and the molecular and genetic basis of its occurrence has been associated with remarkable accuracy to mutations to few nuclear genes such as the melanocortin‐1‐receptor (Mc1r) and agouti signalling protein (ASIP) (Majerus and Mundy 2003; Kingsley et al. 2009; Caro and Mallarino 2020). Therefore, as well as for humans, the role of melanin‐based dark colorations (associated with increased eumelanin accumulation, rather than pheomelanin that imparts yellowish/reddish colour) (Thody et al. 1991) in mammals are associated with increased protection against oxidative stress caused by high UV radiation, which is more intense in tropical regions where melanism in mammals is most frequent (Jablonski and Chaplin 2000; Caro and Mallarino 2020). If, on one hand, the major evolutionary forces driving the evolution of melanism in homeotherms are generally understood, for reptiles, it appears particularly enigmatic because hypotheses have been proposed to explain its adaptive significance and persistence in wild populations. Indeed, it has been reported in most reptile clades, from chelonians (Tucker et al. 1995), to lizards (Recknagel et al. 2018; Dadda et al. 2023) and snakes (Andrén and Nilson 1981; Strugariu and Zamfirescu 2009; Bury et al. 2020) and its occurrence within species can be highly variable at a geographical scale (Castella et al. 2013; Storniolo et al. 2023). However, despite being broadly common among reptiles, the evolution and significance of melanism have not been clarified neither from the theoretical perspective, that is, what are its adaptive drivers and the evolutionary patterns, nor from the molecular one, that is, what is its molecular background. A first hypothesis for the evolution of melanin‐based colorations in ectotherms based on Bogert's rule (Bogert 1949) and was further revised and developed by Clusella Trullas et al. (2007). Bogert's rule states that the frequency of darker animals is higher in colder environments due to thermoregulatory advantage of dark coloration (Bogert 1949). According to this ecogeographic rule, melanistic individuals, being darker, can significantly increase the amount of absorbed incoming light radiations and are therefore expected to be advantaged at low temperature conditions because they can heat up faster than lighter individuals (‘Thermal Melanism Hypothesis’—TMH; Clusella Trullas et al. 2007). Eventually, this results in selective advantages in terms of reproductive success (Andrén and Nilson 1981), immunocompetence (Huyghe et al. 2010; Goldenberg et al. 2022), performance (Zamora‐Camacho et al. 2015) which maintain the occurrence of melanism in populations over time. This hypothesis has been formerly supported mostly by empirical evidence concerning insects (Kingsolver 1987; Pereboom and Biesmeijer 2003); nevertheless, it has been shown to hold true for some reptiles as well (Gibson and Falls 1979; Bittner et al. 2002), indicating that melanism is broadly more common in cooler areas and that melanistic species occur more frequently in such areas than light ones, resulting in greater fitness in sub‐optimal environments. With this respect, a recent work by Martínez‐Freiría et al. (2020) has highlighted that melanisation in the dorsal zig‐zag patterns of Eurasian vipers can play a major role in thermoregulation in cold environments. Nevertheless, in their work, the authors have not considered entirely black individuals (hypermelanism) but rather have focused on those vipers exhibiting disruptive patterns, leaving the question partly unresolved. Up to date, there is no research that has addressed this matter at a larger scale concerning Viperidae as a whole, given that most research was focused on a single target species.

If we were to consider exclusively the putative advantages associated with melanistic phenotypes across reptiles, such as efficiency in thermoregulation, immunocompetence and reproductive output, it would be reasonable to expect that melanism would be more common than observed; therefore, opposite selective forces should be at play to keep its frequency relatively low in populations, especially those under climate‐mediated selective pressure. Indeed, empirical evidence has shown that, despite being associated with increase in females reproductive success over time in *Vipera berus* , melanistic adders tend to suffer from higher predation attempts by avian predators with respect to normally coloured ones (those showing a disruptive pattern on the back) (Andrén and Nilson 1981; Forsman 1995). Consequently, the persistence of this trait in wild populations can be regarded as a context‐dependent phenotype, which is counter‐selected when climatic conditions are favourable and is maintained when adaptive advantages counterbalance the trade‐off.

Among snakes, melanism is equally common with geographical patterns that appear to be rather difficult to untangle. Indeed, it has been detected in some major families such as Colubridae (Bittner et al. 2002; Storniolo et al. 2023) and Viperidae (Andrén and Nilson 1981; Castella et al. 2013). Evidence suggests that melanism might play a significant role in thermal efficiency, growth rate and thus fitness (Andrén and Nilson 1981; Bittner et al. 2002; Castella et al. 2013) when climatic conditions are stringent for ectotherms; however, contrasting results have been drawn for some species inhabiting regions where, presumably, climate is not the major selective force guiding the evolution of dorsal colorations (Tanaka 2007, 2009; Bonnet et al. 2008). Concerning vipers instead, hypermelanism is expected to be increasingly common only when climatic conditions render this trait favourable via a trade‐off process between increased thermal efficiency and predation risks (Allsteadt et al. 2006; Martínez‐Freiría et al. 2017; Chan et al. 2022; Storniolo et al. 2023) irrespective of the phylogenetic relationships as well as of their geographical distribution. Thus, Viperids are an excellent model system to examine how melanism evolved and if it has any adaptive significance. Hence, in this work, we have thoroughly assessed three aspects: (i) the occurrence of melanism in Viperidae across the world; (ii) the correlation between climatic features such as temperature and precipitation with the presence of melanism among vipers in the framework of Bogert's rule; and (iii) the evolutionary history of melanism in the family via ancestral state reconstruction and estimation of phylogenetic signal of the trait.

## 2 Materials and Methods

### 2.1 Data Collection

Data were obtained from the online open access database of iNaturalist (https://www.inaturalist.org), a citizen science initiative promoted by the California Academy of Sciences alongside the National Geographic Society with the aim to make accessible to anybody and anywhere data concerning any type of life on Earth in terms of species spatial and temporal distribution. From this inventory, we queried the 206 viperid species that are mapped on the phylogenetic tree by Zheng and Wiens (2016) and selected those that were recorded in the range of 15–50 records (this thresholds was chosen in accordance with the average occurrence of polymorphisms in wild populations, generally 2%–6%); subsequently, we excluded those species whose coordinates were not available due to conservation measures (a rule that is applied according to IUCN conservation status worse than Near Threatened) and with an error in location accuracy < 1000 m. One last selection step involved picture quality: Those records that either lacked photographic information or whose picture quality was too low (e.g., in the case of low resolution, poor light exposure, moults) were not included in the analysis. By doing so, we thinned the sample to 139 species. For species with more than 50 observations, a subset of 50 records was collected. Prior to analysis, all records were carefully checked for correct species identification by consulting field guides according to species locality, and the occurrence of hypermelanism, intended as a completely black dorsal coloration (no pattern or ornamentation could be visible and phenotype assessment was not hindered by any mean), was assessed by counting the amount of melanistic individuals over the total for each species. Picture selection was carried out by the authors to minimise biases related to picture quality (poor lighting, small proportion of the animal visible, picture taken after manipulation or in hand). By doing so, we were able to select for each species a proper number of records to describe thoroughly the occurrence of melanism at the species level. Overall, we processed a total of 5830 observations with an average of 42 (15–50) records/species.

For widely distributed species, we segmented the geographical occurrences of records in 30 sections of equal longitude (or latitude when the range had a predominantly North‐to‐South extension) and sampled 50 occurrences to cover as evenly as possible the range of the species and the climatic conditions experienced by the species to minimise any potential geographical bias. For each record, we extracted georeferenced climatic data from CRU TS v4.02 bioclimatic maps (https://catalogue.ceda.ac.uk/uuid/b2f81914257c4188b181a4d8b0a46bff/) (University of East Anglia Climatic Research Unit 2020).

From this database, we collected information about three climatic variables: ‘dtr’ (diurnal temperature range expressed in °C), ‘pre’ (precipitation expressed in mm of rain per month) and ‘tmp’ (monthly average daily mean temperature expressed in °C); these weather features were chosen as thermal regimes (tmp and dtr) alongside precipitation frequency (pre) can significantly alter the thermal suitability for ectotherms and therefore are potentially strongly involved in selective processes on phenotypic variability following Bogert's rule. Subsequently, we calculated the average for each species of each variable to test for climatic drivers of the occurrence of melanism. For subsequent analyses, we filtered the global dataset by excluding those records that lacked climatic data; from this thinning, a total of 609 records were eliminated across the dataset and no species was removed, resulting in a total of 5291 records (see Table S1 in Supporting Information for the complete list of the sampled species and Table S2 for those that showed melanism).

### 2.2 Statistical Analyses

We investigated the evolution of dorsal melanism under a comparative approach using the phylogenetic tree by Alencar et al. (2016), calibrated on 11 mitochondrial and nuclear genes. We implemented stochastic character mapping to investigate the evolution of melanism across the phylogeny of vipers according to Pizzigalli et al. (2020). We fitted four potential models of discrete character evolution (defined as a two‐state trait classified as 1 = non‐melanistic and 2 = melanistic) via the *fitDiscrete* function of the ‘geiger’ package (Pennell et al. 2014): Equal Rates (ER—all transition rates are governed by a single parameter), Symmetric (SYM—forward and reverse transitions are regulated by the same parameter), Al‐ Rates‐Differ (ARD—each rate is a unique parameter) and meristic (stepwise occurrence of transitions). Thus, we compared four time‐homogeneous trait evolution models through their respective Akaike Information Criterion corrected for small sample size (AICc) to determine the best fitting model with the function *aicw* from the same package. With this method, we identified that ER, meristic and SYM models performed equally (AICc = 160.7096; ΔAICc = 4.904, w = 0.073) while the ‘best’ fitting was the ARD model (AICc = 155.3213; w = 0.853); hence, we performed ancestral state reconstruction in an ARD framework with the *corHMM* function implemented in the ‘corHMM’ package (Beaulieu and O'Meara 2014) (nstarts = 5000). Consequently, stochastic mapping on the tree was carried out with the makeSimmap function (nSim = 5000), which computes, from the tip states and considering different transition rates from the ARD model, the probabilities of each internal node to be in each state to indicate the average amount of transitions between states and the relative time spent on each state. Furthermore, we investigated whether melanism was driven by phylogenetic signal across Viperidae by computing the *D* statistic. It measures the tendency of closely related taxa to resemble each other by calculating the sum of changes in estimated node trait values along edges in a phylogenetic tree (*D*), where *D* = 0 identifies trait evolution under Brownian motion, and *D* = 1 identifies phylogenetic randomness (Orme et al. 2023). According to phylogeny size, *D* can be significantly variable, and values lower than 0 (highly conserved) or larger than 1 (overdispersed) can be possible. Phylogenetic signal was tested with the *phylo.d* function (5000 repetitions) of the ‘caper’ package (Orme et al. 2023).

To model the probability of a species to be melanistic (dependent variable, 1 = non‐melanistic vs. 2 = melanistic) according to average weather conditions and geographical coordinates, we performed a Phylogenetic Generalised Linear Model with 10,000 independent bootstrap replicates, which incorporates the phylogenetic correlation among species through the *phyloglm* function of the ‘phylolm’ package (Ho and Ané 2014). Prior to analyses, we checked for correlation among predictors. All predictors were significantly correlated with one another (Table S2 of Supporting Information; all *p* < 0.0001) however, given that correlation coefficients were moderate (*ρ* < |0.63| in all cases), climatic variables were directly implemented as fixed effects to model the probability of showing melanism in relation to weather. All analyses were carried out in R 4.2.2 (R Core Team 2022).

## 3 Results

### 3.1 Phylogenetic and Geographical Distribution of Melanism

**FIGURE 1:**
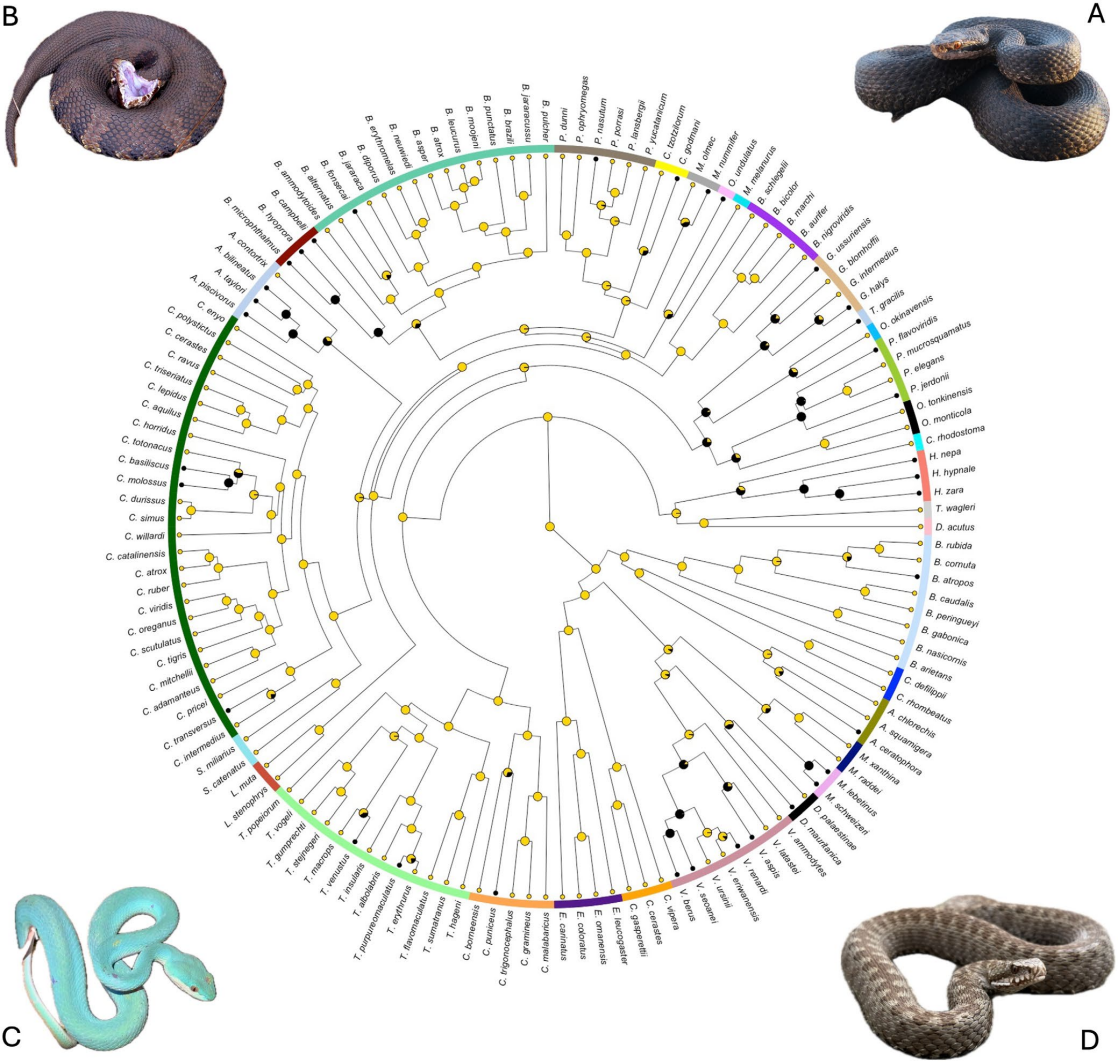
Ancestral state reconstruction on the phylogenetic tree by Alencar et al. (2016), performed using an All Rates Differ model. Pies indicate the probability of each internal or tip node to be in each state (yellow = non‐melanistic, black = melanistic). Representatives of major viper evolutionary lineages are reported in panels: (A) *Gloydius halys* , representative of Central Asian pit vipers; (B) *Agkistrodon piscivorus* , representative of North American pit vipers; (C) *Trimeresurus insularis* , representative of Southeast Asian pit vipers; (D) *Vipera berus* , representative of European vipers.

**FIGURE 2:**
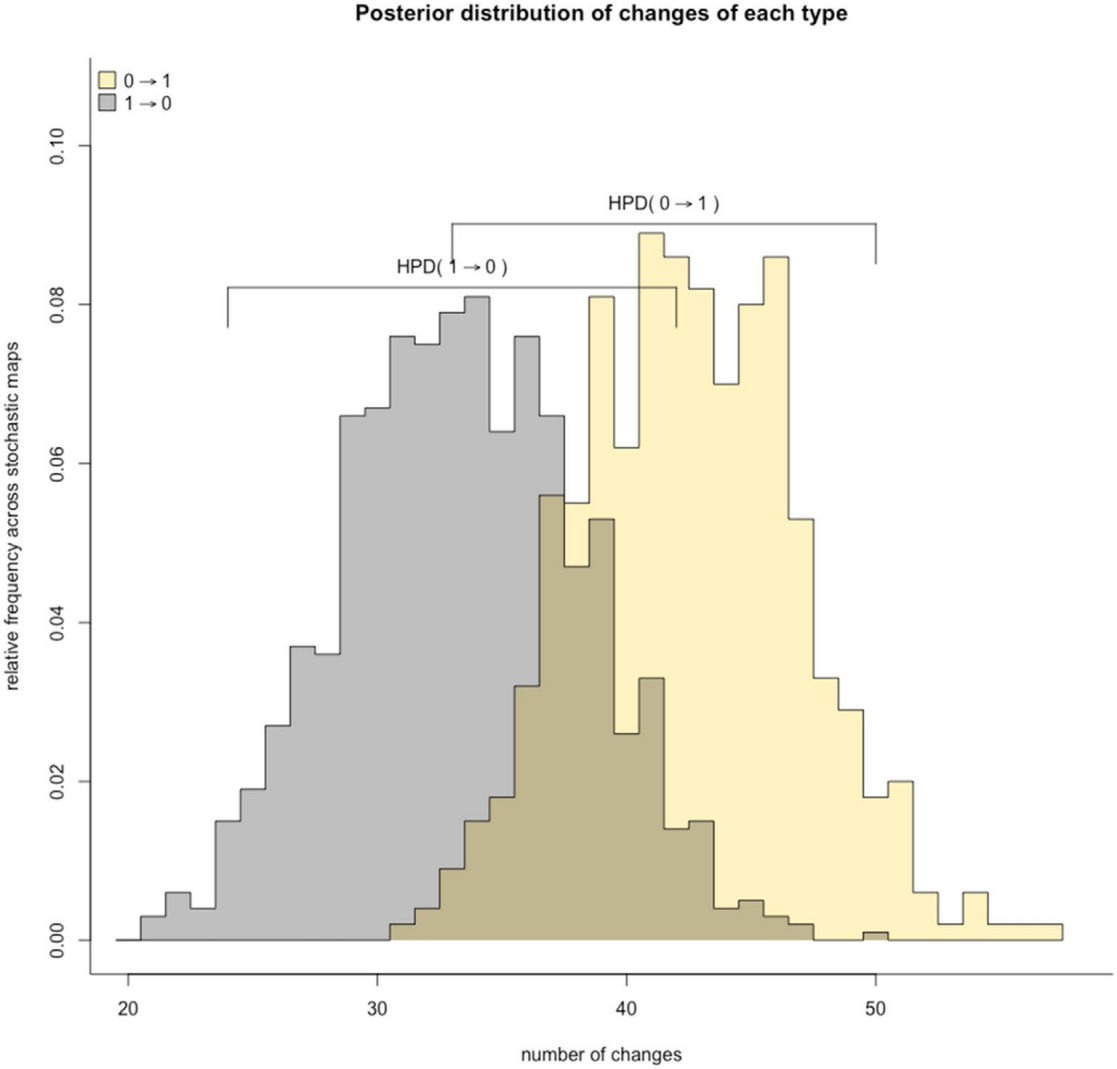
Posterior distribution of changes from one state to the other and vice versa. Each colour represents the starting state of transitions (yellow = non‐melanistic; black = melanistic). Ancestral state reconstruction indicates non‐melanistic as the ancestral phenotype.

Among the 139 species included in our sample, 34 (23.7%) show at least one occurrence of melanism, while the remaining 105 species show only non‐melanistic colorations (globally 128 out of 5830 records were melanistic, 2.2%). Melanism is generally widespread across Viperidae and all continents (see Figure S1). Ancestral state reconstruction, according to the ARD model, indicates that the ancestral state at the root of the tree is the ‘non‐melanistic’ form with a posterior probability of 100% (Figure 1). Across the 5000 generated trees with mapped discrete character states, we estimate on average 76 transitions between states (44–109), respectively, 43 (29–58) from non‐melanistic to melanistic and 33 (15–51) from melanistic to non‐melanistic. The relative amounts of time spent on states 1 and 2 are, respectively, 78.5% and 21.5% (Figure 2). Across the tree, we detected a moderate phylogenetic signal (*D* = 0.57) that indicates both departure from phylogenetic randomness (*p* = 0.004) and from Brownian trait evolution (*p* = 0.017).

### 3.2 Microclimatic Determinants of Melanism

**FIGURE 3:**
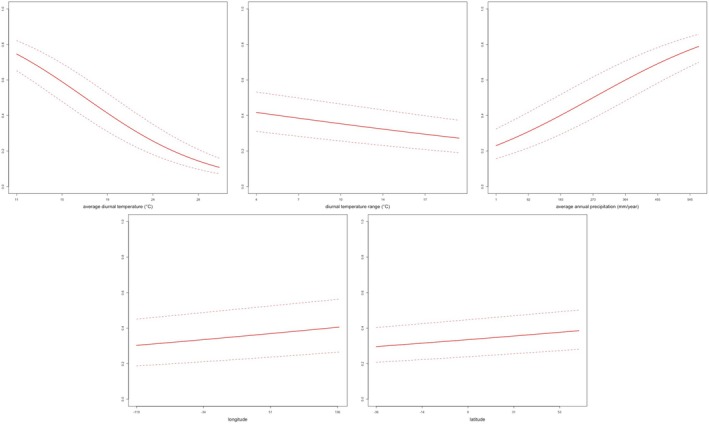
Effects of the climatic variables (average diurnal temperature = tmp, diurnal temperature range = dtr, precipitation = pre from left to right) that were implemented in the phylogenetic generalised linear model (GLM) to account for the effect of climate on the occurrence of melanism across Viperidae. No significant effect was found for dtr and pre, while tmp was negatively correlated with melanism, so that it is more frequent in colder environments, supporting the ‘thermal melanism hypothesis’. Black dots correspond to melanistic species, yellow dots to non‐melanistic ones.

When considering the weather, we did not detect any statistically significant association between diurnal temperature range, precipitation and geographical coordinates and the probability of a species to show melanism; average diurnal temperature was significantly correlated with melanism with a negative effect (*β*_tmp_ = −0.715, SE = 0.229, *z* = −1.23, *p* = 0.001; Table 1), suggesting that melanism occurs more frequently in colder habitats (Figure 3).

**TABLE 1:** Table of the phylogenetic generalised linear model performed on all species to test the effect of climate (average daily temperature = tmp; diurnal temperature range = dtr; precipitation = pre) as well as of geography on the probability of vipers to shown melanism.

	Estimate	Standard error	Lower boot ci	Upper boot ci	*p*
Intercept	−0.640	0.509	−1.532	0.409	0.208
tmp	−0.646	0.229	−1.302	−0.277	**0.001**
dtr	−0.134	0.236	−0.656	0.373	0.096
Pre	0.402	0.241	−0.073	1.015	0.567
Latitude	0.090	0.241	−0.434	0.632	0.708
Longitude	0.149	0.324	−0.371	0.769	0.645

*Note:* Fixed effects were scaled prior to analyses. Statistically significant effects are in bold. 95% bootstrap confidence intervals are shown for each fixed effect.

## 4 Discussion

In the context of body colorations, large effort has been addressed to the understanding of the ecological function of melanism in ectotherms, which has been hypothesised to be tightly related to the thermal efficiency of an animal in the framework of Bogert's rule (Clusella Trullas et al. 2007). Although vipers are known to show highly variable colour and ornamentation patterns (Farallo and Marcos 2009; Martínez‐Freiría et al. 2017; Pizzigalli et al. 2020), melanism in this group of snakes has been investigated scantly and only concerning single species (Andrén and Nilson 1981). With this study, we broadened the scope of previous works and investigated the occurrence of melanism across the whole Viperidae family, both considering the potential effects of phylogeny and ecology. We found that the evolutionary history of melanism was best described by the ARD model, in which transitional rates between states are variable across the phylogeny at each internal node, and the most probable ancestral state of vipers is non‐melanistic. Additionally, the absence of a strong phylogenetic signal of the melanistic trait suggests that this character evolved in multiple independent events along the evolution of vipers and is not maintained over time. With this respect, when accounting for the climatic effects, the negative relation between average daily temperatures and melanism occurrence suggests that it is more frequent in colder environments (Figure 3); however, the absence of significant effects of climate on the frequency of melanism in melanistic species suggests that, presumably, the relative abundance of this trait in wild populations is less influenced by macroevolutionary processes and rather associated with life‐history traits of the species (such as size and consequently body surface/volume ratio). Coherently with the ancestral state reconstruction, the phylogenetic model supports the hypothesis that melanism is a generally rare trait that is likely under negative selection but that, when specific conditions are met (i.e., sub‐optimal climate), it can have significant effects on thermal efficiency compared to non‐melanistic morphs, and thus on fitness. Such scarcity in the occurrence of melanism is presumably affected by higher predation risks, thus decreasing fitness and survivorship, as it was experimentally shown for the adder *Vipera berus* (Andrén and Nilson 1981), where melanistic coloured models suffered significantly higher predation than normally coloured ones. In this work, the ecological significance of melanism is discussed: Melanistic squamates can benefit from advantages in thermoregulation under specific conditions compared to normally coloured ones (e.g., under low thermal regimes; Bittner et al. 2002; Clusella‐Trullas et al. 2008) and can grow larger and have increased reproductive outputs, with major advantages in terms of fitness. The putative advantages associated with melanisation can also involve eco‐physiological aspects such as higher immune responses and lower parasite loads, as well as increased performance (Plasman et al. 2015; Delhey 2019) or protection from incoming UV radiation and the reduction of the associated oxidative stress to DNA (Häkkinen et al. 2002; Rautio and Korhola 2002; Reguera et al. 2014). Therefore, when maximised as in the case of hypermelanism we addressed, fitness is expected to be further boosted. These aspects, though, should not be neglected but were not explored in this work and thus represent an open field for investigation in future research.

The advantages of melanisation emerge only when climate is the limiting factor for reptiles fitness; consistently, the case of the adders from Sweden (Andrén and Nilson 1981) is an example of the benefits of melanisation in vipers at lower average temperatures. The acquisition of hypermelanism implies the loss of wild‐type dorsal patterns and can hamper the functions played by such patterns; Valkonen et al. (2011) addressed the role of zig‐zag patterns of Eurasian vipers to avoid predation and found that they significantly reduce predation attempts, suggesting that they convey an aposematic signal.

Expression of melanism compared to normal colourations implies energetic costs that can have an impact on fitness. This aspect was addressed by Roff and Fairbairn (2013) and Scriber (2020) on insects, indicating that the expression of melanism can even significantly affect fitness by hampering early reproduction as well as influencing behaviour and genetic compatibility. The costs of melanism in endotherms have been explored especially in birds, where melanistic colourations frequently convey signals of quality, dominance and immunocompetence (Roulin and Dijkstra 2003); by manipulating the melanocortin system it was found that an increase in MC_1_ receptor, which results in higher melanogenesis, also correlates with lower adipose tissue and body weight and thus might influence key life‐history aspects (Roulin and Ducrest 2011). Similar constraints of melanisation, although never detected before in snakes, should not be neglected and deserve to be accounted for to evaluate the evolutionary significance of this trait. Consequently, the evolution of melanism in vipers should be regarded as a trade‐off process between thermoregulation advantages and negative effects such as predation risk, lower reproductive efficiency and energy allocation towards colour production rather growth. More explicitly, under ‘normal’ conditions (i.e., non‐stringent climates), the increase in detectability by predators acts against the conservation and spread of such traits among generations, leading to its loss except when adaptation to specific climatic conditions overcomes the selective pressure of predation, leading to the mosaic‐like pattern we observed across vipers' phylogeny.

Hence, although there is scientific evidence at least at the genus level that melanism correlates with climatic patterns, as for the North American Ratsnakes (Hantak et al. 2022), this work provides further support to Gloger's rule at a broad phylogenetic scale (i.e., family level), highlighting the adaptive role of melanism in large‐sized ectotherms in terms of thermal ecology. Our results are generally in accordance with the findings of Goldenberg et al. (2021), where the authors found that darker integuments positively correlate not only with nocturnal behaviour but also, and most interestingly, with higher elevations where temperatures are generally lower. Likewise, concerning the role of melanin‐based phenotypes, it was shown recently by Martínez‐Freiría et al. (2020) that increased pigmentation in the dorsal zig‐zag pattern of Eurasian vipers is associated with colder environments irrespective of the evolutionary history of the species. Our work is in accordance with what the authors have found and significantly expands the scope of their work to the family of Viperidae as well as taking into consideration hypermelanism, highlighting the ecological significance of melanism in squamates.

Nevertheless, in our work, given the nature of the data we collected, we were not able to identify the sex of each snake, so we were unable to account for possible intersexual patterns of variation in the occurrence of melanism across species. This task should be of key importance in future studies when trying to investigate the adaptive significance of such a trait in the perspective of the differential effect of such a trait in males against females.

In conclusion, we believe that melanism should not be regarded as a common trait in Viperidae, but rather as a context‐dependent adaptation that evolves and is maintained in populations under specific climatic conditions. Although Viperidae appear to be excellent models to address this major issue in reptile ecology, the problem is still partly unravelled because global‐scale investigations extending this approach to squamates in their entirety, also concerning the physiological differences between melanistic and non‐melanistic forms, are essential to support definitively the thermal advantage hypothesis.

## Author Contributions

**Roberto Sacchi:** conceptualization (equal), data curation (supporting), formal analysis (supporting), supervision (equal), writing – review and editing (equal). **Stefano Scali:** supervision (equal), writing – review and editing (equal). **Marco Mangiacotti:** data curation (supporting), formal analysis (supporting), supervision (equal), writing – review and editing (equal). **Marco A. L. Zuffi:** supervision (equal), writing – review and editing (equal). **Federico Storniolo:** conceptualization (equal), data curation (lead), formal analysis (lead), investigation (lead), visualization (lead), writing – original draft (lead), writing – review and editing (equal).

## Conflicts of Interest

The authors declare no conflicts of interest.

## Supporting information


Data S1.

## Data Availability

The data that support the findings of this study are openly available in Figshare at https://doi.org/10.6084/m9.figshare.26317678.v4.
