# Supplementary material for: Evolutionary History and Climatic Correlates of Hypermelanism in Viperidae
Source: Ecol Evol. 2025 Apr 16;15(4):e71305. doi: 10.1002/ece3.71305 (PMC12000682; doi:10.1002/ece3.71305)
Supplement: Supplementary file 1 — Data S1. [file ECE3-15-e71305-s001.docx]

**Supplementary Materials of the Paper
"Evolutionary history and climatic correlates of hypermelanism in Viperidae"**

In the following document, are presented additional elements containing the complete table of the sampled species, for each we show the continent, how many pictures were processed and how many of them were melanic. Additionally, we show the table of correlation between predictors implemented in the phylogenetically informed GLM (*phyloglm*) and the distribution map of all record, discriminated by phenotype.


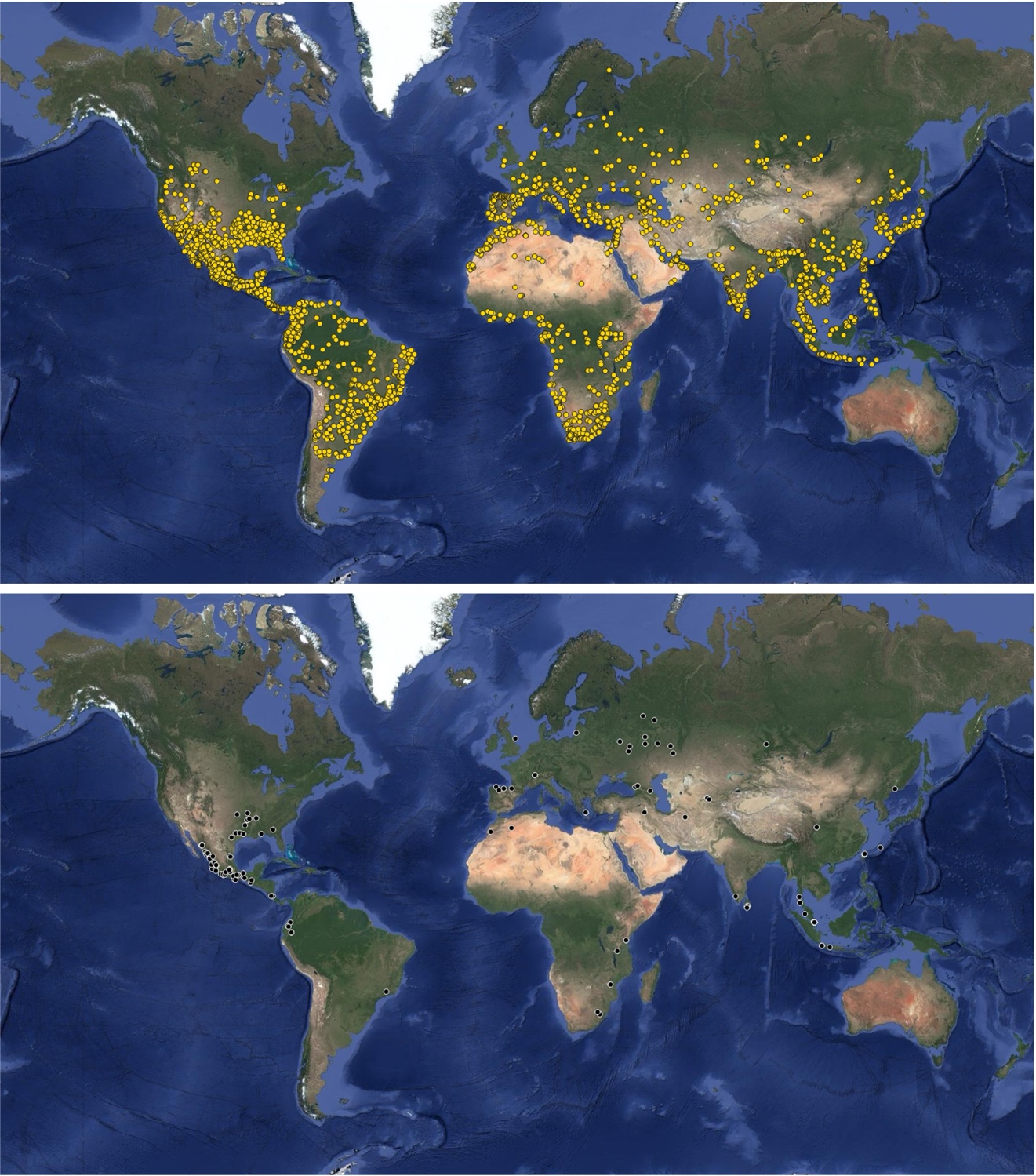


Figure S1. Distribution of all records we processed across the 139 sampled species of Viperidae. in the top panel (A) are shown non-melanic individuals, in the bottom panel (B) are shown the melanic individuals.

| **Species** | **Pictures** | **Melanic** |
| --- | --- | --- |
| *Agkistrodon bilineatus* | 35 | 25 |
| *Agkistrodon contortrix* | 49 | 0 |
| *Agkistrodon piscivorus* | 49 | 12 |
| *Agkistrodon taylori* | 23 | 3 |
| *Atheris ceratophora* | 16 | 3 |
| *Atheris chlorechis* | 20 | 0 |
| *Atheris squamigera* | 40 | 0 |
| *Bitis arietans* | 47 | 0 |
| *Bitis atropos* | 43 | 4 |
| *Bitis caudalis* | 49 | 0 |
| *Bitis cornuta* | 21 | 0 |
| *Bitis gabonica* | 47 | 0 |
| *Bitis nasicornis* | 34 | 0 |
| *Bitis peringueyi* | 49 | 0 |
| *Bitis rubida* | 36 | 0 |
| *Bothriechis aurifer* | 27 | 0 |
| *Bothriechis bicolor* | 17 | 0 |
| *Bothriechis marchi* | 24 | 0 |
| *Bothriechis nigroviridis* | 41 | 0 |
| *Bothriechis schlegelii* | 44 | 0 |
| *Bothrocophias campbelli* | 31 | 1 |
| *Bothrocophias hyoprora* | 34 | 2 |
| *Bothrocophias microphthalmus* | 48 | 1 |
| *Bothrops alternatus* | 49 | 0 |
| *Bothrops ammodytoides* | 48 | 0 |
| *Bothrops asper* | 43 | 0 |
| *Bothrops atrox* | 47 | 0 |
| *Bothrops brazili* | 19 | 0 |
| *Bothrops diporus* | 45 | 0 |
| *Bothrops erythromelas* | 23 | 0 |
| *Bothrops fonsecai* | 23 | 1 |
| *Bothrops jararaca* | 49 | 0 |
| *Bothrops jararacussu* | 41 | 0 |
| *Bothrops leucurus* | 36 | 0 |
| *Bothrops moojeni* | 43 | 0 |
| *Bothrops neuwiedi* | 43 | 0 |
| *Bothrops pulcher* | 14 | 0 |
| *Bothrops punctatus* | 35 | 0 |
| *Calloselasma rhodostoma* | 45 | 0 |
| *Causus defilippii* | 42 | 0 |
| *Causus rhombeatus* | 49 | 0 |
| *Cerastes cerastes* | 44 | 0 |
| *Cerastes gasperettii* | 49 | 0 |
| *Cerastes vipera* | 29 | 0 |
| *Cerrophidion godmani* | 42 | 2 |
| *Cerrophidion tzotzilorum* | 22 | 0 |
| *Craspedocephalus borneensis* | 16 | 0 |
| *Craspedocephalus gramineus* | 44 | 0 |
| *Craspedocephalus malabaricus* | 45 | 0 |
| *Craspedocephalus puniceus* | 42 | 2 |
| *Craspedocephalus trigonocephalus* | 44 | 0 |
| *Crotalus adamanteus* | 49 | 0 |
| *Crotalus aquilus* | 41 | 0 |
| *Crotalus atrox* | 50 | 0 |
| *Crotalus basiliscus* | 41 | 1 |
| *Crotalus catalinensis* | 28 | 0 |
| *Crotalus cerastes* | 48 | 0 |
| *Crotalus durissus* | 47 | 0 |
| *Crotalus enyo* | 33 | 0 |
| *Crotalus horridus* | 48 | 0 |
| *Crotalus intermedius* | 45 | 0 |
| *Crotalus lepidus* | 46 | 0 |
| *Crotalus mitchellii* | 37 | 0 |
| *Crotalus molossus* | 47 | 1 |
| *Crotalus oreganus* | 46 | 0 |
| *Crotalus polystictus* | 46 | 0 |
| *Crotalus pricei* | 47 | 0 |
| *Crotalus ravus* | 47 | 0 |
| *Crotalus ruber* | 45 | 0 |
| *Crotalus scutulatus* | 48 | 0 |
| *Crotalus simus* | 48 | 0 |
| *Crotalus tigris* | 48 | 0 |
| *Crotalus totonacus* | 38 | 0 |
| *Crotalus transversus* | 35 | 0 |
| *Crotalus triseriatus* | 48 | 2 |
| *Crotalus viridis* | 48 | 0 |
| *Crotalus willardi* | 44 | 0 |
| *Daboia mauritanica* | 35 | 0 |
| *Daboia palaestinae* | 39 | 2 |
| *Deinagkistrodon acutus* | 47 | 0 |
| *Echis carinatus* | 38 | 0 |
| *Echis coloratus* | 15 | 0 |
| *Echis leucogaster* | 25 | 0 |
| *Echis omanensis* | 45 | 0 |
| *Gloydius blomhoffii* | 41 | 0 |
| *Gloydius halys* | 47 | 0 |
| *Gloydius intermedius* | 44 | 1 |
| *Gloydius ussuriensis* | 44 | 0 |
| *Hypnale hypnale* | 41 | 1 |
| *Hypnale nepa* | 8 | 1 |
| *Hypnale zara* | 28 | 1 |
| *Lachesis muta* | 46 | 1 |
| *Lachesis stenophrys* | 28 | 0 |
| *Macrovipera lebetinus* | 47 | 0 |
| *Macrovipera schweizeri* | 21 | 7 |
| *Metlapilcoatlus nummifer* | 38 | 1 |
| *Metlapilcoatlus olmec* | 25 | 0 |
| *Mixcoatlus melanurus* | 13 | 0 |
| *Montivipera raddei* | 14 | 0 |
| *Montivipera xanthina* | 40 | 0 |
| *Ophryacus undulatus* | 27 | 0 |
| *Ovophis monticola* | 32 | 0 |
| *Ovophis okinavensis* | 21 | 0 |
| *Ovophis tonkinensis* | 25 | 0 |
| *Porthidium dunni* | 40 | 0 |
| *Porthidium lansbergii* | 50 | 0 |
| *Porthidium nasutum* | 46 | 2 |
| *Porthidium ophryomegas* | 42 | 0 |
| *Porthidium porrasi* | 22 | 0 |
| *Porthidium yucatanicum* | 39 | 0 |
| *Protobothrops elegans* | 23 | 0 |
| *Protobothrops flavoviridis* | 28 | 0 |
| *Protobothrops jerdonii* | 21 | 0 |
| *Protobothrops mucrosquamatus* | 48 | 0 |
| *Sistrurus catenatus* | 49 | 0 |
| *Sistrurus miliarius* | 50 | 0 |
| *Trimeresurus albolabris* | 49 | 0 |
| *Trimeresurus erythrurus* | 34 | 0 |
| *Trimeresurus flavomaculatus* | 25 | 0 |
| *Trimeresurus gracilis* | 35 | 5 |
| *Trimeresurus gumprechti* | 20 | 0 |
| *Trimeresurus hageni* | 45 | 0 |
| *Trimeresurus insularis* | 43 | 0 |
| *Trimeresurus macrops* | 49 | 0 |
| *Trimeresurus popeiorum* | 48 | 0 |
| *Trimeresurus purpureomaculatus* | 38 | 25 |
| *Trimeresurus stejnegeri* | 49 | 0 |
| *Trimeresurus sumatranus* | 20 | 0 |
| *Trimeresurus venustus* | 32 | 1 |
| *Trimeresurus vogeli* | 48 | 0 |
| *Tropidolaemus wagleri* | 46 | 0 |
| *Vipera ammodytes* | 45 | 0 |
| *Vipera aspis* | 45 | 2 |
| *Vipera berus* | 47 | 9 |
| *Vipera eriwanensis* | 15 | 0 |
| *Vipera latastei* | 44 | 0 |
| *Vipera renardi* | 45 | 5 |
| *Vipera seoanei* | 46 | 4 |
| *Vipera ursinii* | 48 | 0 |
| **Total** | **5291** | **128** |

Table S1. Table of all the species involved in this study, indicating the total amount of pictures sampled per species and how many were melanic.

|  | **pre** | **dtr** | **tmp** | **lon** | **lat** |
| --- | --- | --- | --- | --- | --- |
| **pre** | 1 |  |  |  |  |
| **dtr** | -0.556 | 1 |  |  |  |
| **tmp** | 0.405 | -0.181 | 1 |  |  |
| **lon** | 0.265 | -0.626 | 0.128 | 1 |  |
| **lat** | -0.340 | 0.173 | -0.259 | -0.097 | 1 |

Table S2. Table of the correlation coefficients among climatic predictors; all correlations were statistically significant (P < 0.0001 in all cases), however given that correlations were moderate (ρ < |0.626| in all cases), all were implemented as fixed effects in both *phyloglm* and *phylolm*.
